# Getting Back to the Basics of Translational Research

**DOI:** 10.1371/journal.ppat.1005534

**Published:** 2016-07-21

**Authors:** Paul M. Lieberman

**Affiliations:** Gene Expression and Regulation Program, The Wistar Institute, Philadelphia, Pennsylvania, United States of America; The Fox Chase Cancer Center, UNITED STATES

How do viruses cause cancer? As a naïve graduate student in the years before kits and personal computers, I found myself fascinated by viral “immortalization,” the process by which a virus induces a cell to live forever. The immortalizing virus of interest was named Epstein–Barr Virus (EBV) after its co-discoverers and was isolated from tumors commonly found in African children. EBV was also known to be a major cause of infectious mononucleosis, commonly referred to as the “kissing disease,” and a potential agent in chronic fatigue syndromes. For these reasons, I suspect, EBV was slow to get the respect it deserved. We now know that EBV is found in diverse tumors and is responsible for almost ~200,000 cancer cases each year. At the time, my interests were to understand the viral mechanisms that transform a resting mortal cell to replicate uncontrollably. It was also tempting to speculate over a beer whether viral immortalization could reveal fresh insights into human mortality and life span. On more sober occasions, the question of how a virus could cause cancer seemed a big enough challenge.

Over the years, my interest in viral immortalization morphed into an investigation of viral gene regulation and latency. How does EBV establish long-term latent infection and express only a few essential viral genes necessary to keep the host cell dividing and alive? To get at these questions, we needed to combine many different methods ranging from functional genomics to structural biology, requiring expertise in diverse disciplines far beyond my capabilities. Building a team of collaborators with the appropriate expertise and identifying the most appropriate technologies is essential. Selecting the right tools and team to answer the bigger questions is not always straightforward, but establishing multi-disciplinary collaborations can open many otherwise closed doors.

Among the most important and challenging steps in a research career is identifying a significant long-term research problem. This sounds easy, but it has many snags. Highly ambitious long-term goals, like curing EBV cancers, may not be achievable without more mundane short-term groundwork. And short-term goals, like the need to get the next paper published or grant funded, can sometimes distract from the long-term plan. Working on tangential problems is necessary to overcome obstacles and can lead to new and unanticipated discoveries. But keeping sight of the long-term goal can be a great strength for a research program. If all goes well, the research focus will be aligned with reviewers and funding agencies. Fortunately for EBV research, foundations like Cancer Research UK have recently recognized the eradication of EBV-associated cancers as one of the decade’s grand challenges. So how best to respond to this grand challenge?

From my perspective, studying the basic mechanisms of EBV latency is the best path to identify viral-specific targets for therapeutic intervention. At least half of my group is committed to developing small molecules to either inhibit EBV latency or stimulate viral reactivation, two possible strategies to treat latent viruses in cancer cells. Viral reactivation from latency is attractive because it is likely to trigger the host immune system to eliminate EBV-infected cancer cells. Inhibiting latency should lead to the loss of viral DNA from cancer cells that depend on EBV for growth and survival. In principle, inhibitors of EBV latency or activators of viral lytic antigens should be valuable therapies to treat EBV-associated cancer and related disease.

Of course these more ambitious projects are risky and require vast sums of funding. The pharmaceutical industry and venture capitalists have no tolerance for financing the early stages of long-term, high-risk projects, so public funding of academic investigators will be the only possible path forward. Although there are many academics who consider translational research a corruption of academic freedom, the urgency of real-world unmet medical needs becomes very apparent if one only takes a look at the economic and human cost of disease. It will be essential for academic investigators to fulfill these obligations to society. Ironically, an editorial opposing the funding of basic academic research was recently written by Matt Ridley in *The Wall Street Journal* arguing that public sponsorship of basic research blocks private investment and derails innovation. In fact, the only chance for innovative solutions to long-term challenges will be through public funding of basic academic research. Encouraging scientists to focus on unmet medical needs as well as conceptual advances in basic science should be a high priority for public and private investment in a healthy future.

**Image 1:**
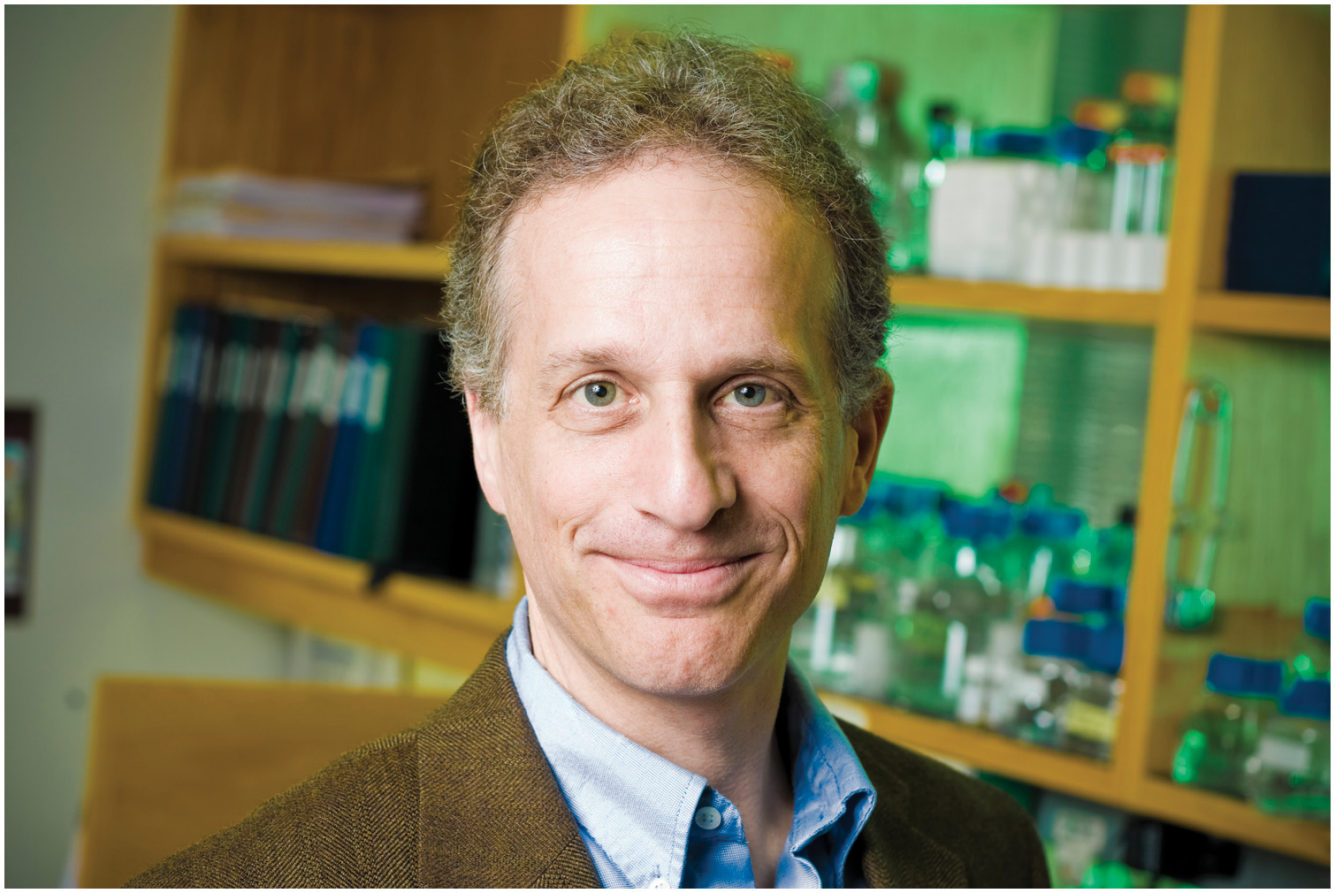
Paul M. Lieberman, Hilary Koprowski Professor, The Wistar Institute.

